# High-fidelity deconvolution for acoustic-resolution photoacoustic microscopy enabled by convolutional neural networks

**DOI:** 10.1016/j.pacs.2022.100360

**Published:** 2022-04-26

**Authors:** Fei Feng, Siqi Liang, Jiajia Luo, Sung-Liang Chen

**Affiliations:** a University of Michigan–Shanghai Jiao Tong University Joint Institute, Shanghai Jiao Tong University, Shanghai 200240, China; b Institute of Medical Technology, Peking University Health Science Center, Beijing 100191, China; c Biomedical Engineering Department, Peking University, Beijing 100191, China; d Peking University People’s Hospital, Beijing 100044, China; e State Key Laboratory of Advanced Optical Communication Systems and Networks, Shanghai Jiao Tong University, Shanghai 200240, China; f Engineering Research Center of Digital Medicine and Clinical Translation, Ministry of Education, Shanghai 200030, China

**Keywords:** Photoacoustic imaging, Deconvolution, Deep learning, High-fidelity deconvolution, Multiscale imaging

## Abstract

Acoustic-resolution photoacoustic microscopy (AR-PAM) image resolution is determined by the point spread function (PSF) of the imaging system. Previous algorithms, including Richardson–Lucy (R–L) deconvolution and model-based (MB) deconvolution, improve spatial resolution by taking advantage of the PSF as prior knowledge. However, these methods encounter the problems of inaccurate deconvolution, meaning the deconvolved feature size and the original one are not consistent (e.g., the former can be smaller than the latter). We present a novel deep convolution neural network (CNN)-based algorithm featuring high-fidelity recovery of multiscale feature size to improve lateral resolution of AR-PAM. The CNN is trained with simulated image pairs of line patterns, which is to mimic blood vessels. To investigate the suitable CNN model structure and elaborate on the effectiveness of CNN methods compared with non-learning methods, we select five different CNN models, while R–L and directional MB methods are also applied for comparison. Besides simulated data, experimental data including tungsten wires, leaf veins, and *in vivo* blood vessels are also evaluated. A custom-defined metric of relative size error (RSE) is used to quantify the multiscale feature recovery ability of different methods. Compared to other methods, enhanced deep super resolution (EDSR) network and residual in residual dense block network (RRDBNet) model show better recovery in terms of RSE for tungsten wires with diameters ranging from 30 μm to 120 μm. Moreover, AR-PAM images of leaf veins are tested to demonstrate the effectiveness of the optimized CNN methods (by EDSR and RRDBNet) for complex patterns. Finally, *in vivo* images of mouse ear blood vessels and rat ear blood vessels are acquired and then deconvolved, and the results show that the proposed CNN method (notably RRDBNet) enables accurate deconvolution of multiscale feature size and thus good fidelity.

## 1 Introduction

Photoacoustic (PA) imaging has the advantage in deep tissue imaging compared with optical imaging and has been demonstrated for multiscale *in vivo* imaging [1]. PA imaging can be implemented for microscopy, termed PA microscopy (PAM), which can be further distinguished into optical-resolution PAM (OR-PAM) and acoustic-resolution PAM (AR-PAM). For OR-PAM, optical focusing is tighter than acoustic focusing, and the lateral resolution is determined by optical focusing and restricted by optical diffraction. On the other hand, for AR-PAM, acoustic focusing is tighter, and the lateral resolution is decided by acoustic focusing and limited by acoustic diffraction. As a result, AR-PAM has an advantage over OR-PAM in deep tissue imaging by taking advantage of the diffused light and deep acoustic penetration [2], [3]. AR-PAM has been successfully applied to microvascular imaging [2], [4], [5].

Lateral resolution of AR-PAM is determined by the center frequency and numerical aperture (NA) of a focused acoustic transducer. A high-frequency and high-NA transducer can be used in AR-PAM to achieve high lateral resolution. However, in this case, it needs to detect high-frequency acoustic waves, which are severely attenuated in biological tissues, in turn hindering deep penetration. Besides, high NA leads to a reduced depth of focus and working distance, which causes difficulties in selected imaging applications. Alternatively, a deconvolution algorithm can be applied to enhance lateral resolution of AR-PAM while circumventing the above-mentioned issues. Enhanced lateral resolution in AR-PAM would benefit applications such as PA velocimetry [6] and disease characterization [7].

Deconvolution algorithms have been used in PA imaging, including PA computed tomography (PACT) [8], [9], [10], OR-PAM [11], [12], [13], and AR-PAM [4], [5], [14], [15]. Different deconvolution algorithms have been tested in AR-PAM, mainly using R–L deconvolution [4], [5] and MB deconvolution [14], [15]. When applying deconvolution, the point spread function (PSF) is usually required as a prior, and the PSF of AR-PAM is determined by the focal zone of the acoustic transducer. Richardson–Lucy (R–L) deconvolution has been widely used for astronomical imaging [16], [17] and has been applied to improve spatial resolution of AR-PAM for both focal and out-of-focus regions [4], [5]. However, R–L deconvolution is an iterative method, and when too many iterations are performed, the deconvolved feature size could be smaller than the original object size, which is not accurate. In addition to R–L deconvolution, the model-based (MB) method is another deconvolution method. MB deconvolution reconstructs the original image based on an optimization approach. The MB method has been used in PACT and AR-PAM to improve spatial resolution [9], [10], [14], [15]. Since both the R–L and MB methods assume that the original image is composed of point-like objects, the processed results suffer from line discontinuities. Previously, we proposed a directional MB (D-MB) algorithm to solve the issue of discontinuity based on one-dimensional (1D) deconvolution along various directions [15], yet neither MB nor D-MB can realize high-fidelity recovery of the multiscale feature size. In summary, the existing deconvolution algorithms have several challenges. First, the reconstructed size can be smaller than the original object size, as mentioned previously. Secondly, for different original object size, the deconvolution algorithms cannot accurately recover multiscale object size. Therefore, an approach needs to be developed to overcome these issues.

**Fig. 1:**
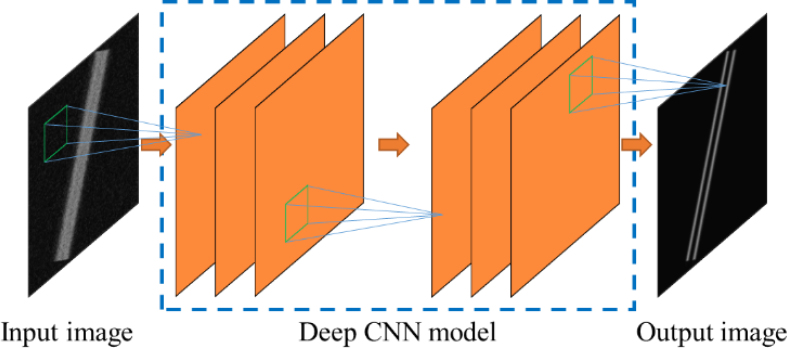
Workflow of the CNN method.

Compared with the above non-learning method, the learning-based method uses a data-driven approach to learn image restoration knowledge. Among them, the deep learning method has attracted wide attention in recent years. Deep learning models (aka deep neural networks) consist of multiple processing layers that learn the complex implicit rule between the input and output with a large amount of data for training [18]. Convolutional neural networks (CNNs), one of implementations of the deep learning, have shown great performance in both natural and biomedical image processing [18], [19], [20]. CNNs have also been applied to PA image processing, such as PACT reconstruction, sparse OR-PAM recovery, and AR-PAM defocusing [21], [22], [23], [24]. To the best of our knowledge, AR-PAM deconvolution by deep learning has not been studied yet. To address the above challenges of existing AR-PAM deconvolution algorithms by deep learning, it is crucial to prepare training data and identify the suitable CNN model structure.

In this work, we investigate CNN-based deconvolution to improve lateral resolution of AR-PAM as well as identify the suitable CNN models to optimize the performance. Five existing CNN models are trained with 1218 simulated AR-PAM image pairs for comparison [24], [25], [26], [27], [28]. These five CNN models are fully dense UNet (FDUNet) [24], residual channel attention network (RCAN) [25], enhance deep super resolution (EDSR) network [26], residual in residual dense block network (RRDBNet) [27], and feature fusion attention network (FFANet) [28]. CNN-based deconvolution to recover the original object size is first confirmed by AR-PAM images of different diameters of tungsten wires. The tungsten wire with a diameter down to ∼30 μm can be accurately recovered, which is less than half of the lateral resolution of 65 μm of the AR-PAM system. Then, AR-PAM images of leaf veins, *in vivo* mouse ear blood vessels, and *in vivo* rat ear blood vessels, all of which display multiscale line branches, are deconvolved by CNN models, and the results show not only high-fidelity recovery of multiscale feature size but also good continuity. Among the five CNN models, EDSR and RRDBNet show advantages over other models in resolution enhancement with high-fidelity recovery of multiscale AR-PAM images.

## 2 Methods

### 2.1 Overall workflow

As shown in Fig. 1, a CNN model is used as a learner to learn a function from the input image to the output image. Here, the input image is a low-resolution PA image and is sent to the deep CNN model. After the CNN processing, the resolution-enhanced PA image is predicted. To learn such knowledge, a ground truth image (i.e., the high-resolution image) is used as guidance to measure the difference between the ground truth PA image and the CNN-predicted PA image. By minimizing the difference, the model weights will be updated and the model will perform better. After the CNN model is well optimized, it is used for resolution enhancement of experimentally-acquired AR-PAM images (including tungsten wires, leaf veins, and blood vessels *in vivo*). As mentioned previously, the training data and the CNN model structure, which are detailed as follows, are important for the overall workflow and deconvolution performance.

### 2.2 Training data generation and evaluation

The training data can be obtained either experimentally or numerically (i.e., synthetic or simulated data). For the experimental method, a high-resolution AR-PAM system is needed to acquire the ground truth PA image. Building the AR-PAM system with high lateral resolution (less than half of the lateral resolution of 65 μm) could be challenging. Therefore, a synthetic method is adopted to generate the training data. In principle, the AR-PAM system can be assumed as a linear spatial shift-invariant system around the focal region and expressed as:

(1) $$o^{\prime }=p⊗o+n,$$

where $o^{\prime }$ represents the acquired PA image, p represents the PSF, o represents the ground truth PA image (i.e., the original object), n represents noise, and ⊗ denotes convolution operation. Since the focal zone of a focused acoustic transducer used in AR-PAM is usually a Gaussian profile, the PSF is assumed to be a Gaussian profile. The Gaussian profile (or Gaussian distribution) can be characterized by parameters of mean and standard deviation, which are denoted as $\mu_{p}$ and $\sigma_{p}$, respectively. If we assume that the ground truth PA image is also a Gaussian profile, which has a mean $\mu_{o}$ and a standard deviation $\sigma_{o}$, the convolution result of them is still is a Gaussian profile, whose mean and standard deviation are denoted as $\mu_{c}$ and $\sigma_{c}$, respectively. Besides, $\sigma_{p}$ and $\sigma_{o}$, and $\sigma_{c}$ are related as follows:

(2) $$\sigma_{c}^{2}=\sigma_{p}^{2}+\sigma_{o}^{2}.$$

**Fig. 2:**
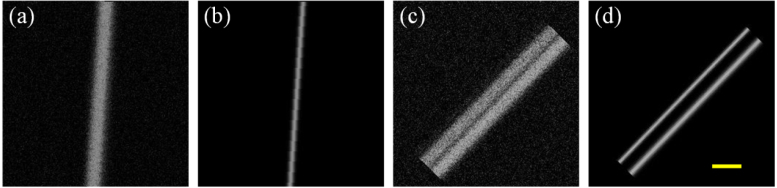
Examples of training data. (a, b) The high-resolution (b) and corresponding low-resolution (a) image pair of a single line. (c, d) The high-resolution (d) and corresponding low-resolution (c) image pair of two closely-located lines. Diameters of the lines are 76μm in (a), 33μm in (b), and 25μm (top) and 33μm (bottom) in (d). Note that due to the added noise, diameters in (c) are no longer resolvable. SNR is 28.49 dB in (a) and 23.27 dB in (c). No noise is added to (b) and (d). Scale bar: 200 μm.

Since the standard deviation of a Gaussian profile is linearly proportional to its full width at half maximum (FWHM), we can further obtain the FWHM relation:

(3) $$d_{c}=\sqrt{d_{p}^{2}+d_{o}^{2}},$$

where $d_{c}$ is the FWHM of the acquired PA image, $d_{p}$ is the FWHM of the PSF, and $d_{o}$ is the FWHM of the ground truth PA image. Then, the training data are generated numerically according to Eq. (1). As microvascular imaging is one of the most common applications of AR-PAM, line patterns are used. As the line pattern has 1D sparsity, the training data is generated by 1D convolution. Two types of line patterns, a single line and two closely-located lines, are used for training, as shown in Fig. 2. The training data consists of high-resolution and corresponding low-resolution image pairs. The low-resolution image is generated by 1D convolution of the high-resolution image (ground truth) along the direction perpendicular to the line. The FWHM of the ground truth ranges from 20 μm to 200 μm. Ideally, if the AR-PAM image is free of noise, the FWHM of the ground truth PA image can be easily extracted using Eq. (3). However, as noise always exists in real PA images, it deteriorates the image quality. Moreover, existing deconvolution methods may begin to fail in high noise environments. To account for noise in our CNN model, some speckle noise and Gaussian noise (n in Eq. (1)) are added to the low-resolution image. The noise is added with *imnoise* in MATLAB. To take a wide range of noise level into consideration, the low-resolution images after adding noise have a SNR distribution of 30.20 ± 16.48 dB (mean ± standard deviation).

With simulated data, the CNN model is trained in a supervised manner. We use L1 loss function for model training, which can be expressed as:

(4) $$Loss=\frac{1}{MN}\overset{M,N}{\underset{m,n}{\sum }}\left|o_{mn}-o_{mn}^{\prime }\right|,$$

where $o_{mn}$ and $o_{mn}^{\prime }$ are the pixel values of the ground truth PA image and predicted PA image on the $m^{th}$ row and $n^{th}$ column, respectively. To evaluate the deconvolution performance, two metrics, peak signal to noise ratio (PSNR) and structural similarity (SSIM) index, are used. Besides, signal to noise ratio (SNR) and contrast to noise ratio (CNR) are used to evaluate noise level and image contrast, respectively. More details about PSNR, SSIM, SNR, and CNR are described in Section 1 of Supplement 1. To evaluate the multiscale feature recovery ability, the relative size error (RSE) is defined in the following equation:

(5) $$RSE=\frac{\left|d_{o^{\prime }}-d_{o}\right|}{d_{o}},$$

where $d_{o^{\prime }}$ is the FWHM of the predicted PA image.

### 2.3 CNN models and training settings

To investigate the effectiveness of different CNN methods, five different model structures are compared, which include FDUNet [24], RCAN [25], EDSR [26], RRDBNet [27], and FFANet [28]. FDUNet is used because it showed good performance in image reconstruction of defocused AR-PAM data [24]. EDSR and RRDBNet are selected since they achieved good performance in super resolution of natural images [26], [27]. Besides, feature dependencies were mined with the design of channel attention in RCAN [25]. Compared with RCAN, non-local attention was exploited with the design of pixel attention in FFANet [28]. By comparing the deconvolution performance of the five representative CNN methods, a more suitable model for AR-PAM image deconvolution can be obtained. More details about these CNNs and training details are described in Section 2 of Supplement 1.

### 2.4 Experiments

The experiments were conducted with both simulated data and experimental data. The AR-PAM system using a focused transducer with a center frequency of 50 MHz, NA of 0.4, and focal length of 6.7 mm was employed. The experimentally-measured PSF was 65 μm (FWHM). Therefore, a 1D Gaussian curve with FWHM of 65 μm was used as the PSF for training data generation. Then, 1218 and 383 image pairs using images of line patterns were generated according to Eq. (1) for model training and testing, respectively. As mentioned above, five CNN models were used. Besides, two traditional methods, R–L deconvolution and D-MB deconvolution, were used for comparison. We first trained different CNN models using the training set of the simulated data and then compared different methods (trained CNN models and traditional methods) using the testing set of the simulated data. Then, the trained CNN models and traditional methods were tested using the experimental data including the phantom and *in vivo* images.

The experimental data were prepared as follows. As for phantom samples, both tungsten wires and Banyan leaves were prepared for imaging. Specifically, tungsten wires with different diameters of 20 μm to 120 μm were prepared. Note that the difference between using the diameter and the FWHM as the original object size for convolution is discussed later. On the other hand, Banyan leaves were immersed in carbon ink for 24 h and dried in an oven for 10 min, and then, the leaves were placed on a glass slide and sealed by silicone. As for the *in vivo* data, blood vessels of both mouse and rat ears were imaged, which is to demonstrate the effectiveness of our method for *in vivo* applications. Specifically, a 6-week-old nude mouse was used. The mouse was anesthetized by a gas anesthetic machine (R500IP, RWD Life Science) with a gas mixture of 1% isoflurane and oxygen. The hairs on the mouse ear were removed with the help of a cleaning cream. During image acquisition, the mouse was fixed on a homemade platform. The optical fluence deposited on the biological tissue was ∼15 $\mathrm{mJ}/{\mathrm{cm}}^{2}$, which is below the American National Standards Institute safety limit (20 $\mathrm{mJ}/{\mathrm{cm}}^{2}$). The sample preparation and image acquisition of the rat experiment were similar to that of the mouse experiment except a gas mixture of 3.5% isoflurane and oxygen used to anesthetize the rat. All experimental animal procedures were implemented in conformity with the laboratory animal protocol approved by the Laboratory Animal Care Committee of Shanghai Jiao Tong University.

**Fig. 3:**
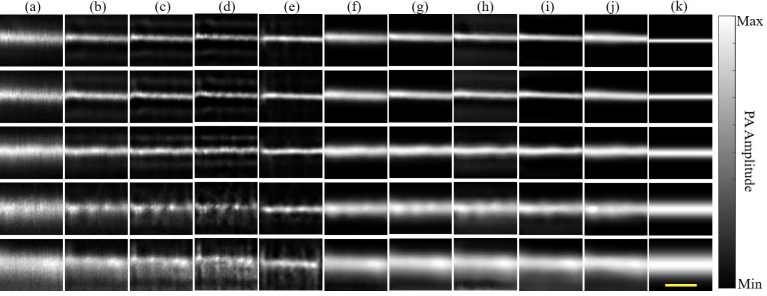
AR-PAM images of tungsten wires: (a) raw PA images, (b) by R-L-10, (c) by R-L-15, (d) by R-L-30, (e) by D-MB, (f) by FDUNet, (g) by RCAN, (h) by EDSR, (i) by RRDBNet, (j) by FFANet, and (k) the ground truth images (simulated using Gaussian profiles with the FWHM equal to the original diameters of tungsten wires). Top to bottom rows correspond to the original diameter of 20 μm, 30 μm, 50 μm, 80 μm, and 120 μm of tungsten wires, respectively. Scale bar: 200 μm.

**Table 1:** Image recovery performance of different methods on the testing set of the simulated data.

Case	PSNR (dB)	SSIM	# of parameters (M)
Raw data	22.01	0.1116	–
R-L-10	29.73	0.2104	–
R-L-15	31.03	0.2866	–
R-L-30	32.94	0.4337	–
D-MB	35.29	0.7396	–
FDUNet	33.40	0.9424	17.90
RCAN	34.80	0.9541	3.05
EDSR	34.88	0.9636	1.52
RRDBNet	36.13	0.9713	1.59
FFANet	36.96	0.9762	1.51

## 3 Results

Table 1 shows the image recovery performance of different methods on the testing set of the simulated data. As R-L deconvolution is an iterative method, three iteration numbers of 10, 15, and 30 are tested, which are denoted as R-L-10, R-L-15, and R-L-30. Table 1 shows that all methods have higher PSNR and SSIM than the raw image. For the R-L method, both PSNR and SSIM increase with the iteration numbers. Further, D-MB achieves higher PSNR and SSIM than R-L methods. More importantly, these five CNN methods realize distinctly higher SSIM compared with R-L and D-MB methods. Besides, the PSNR of these five CNN methods is comparable to that of the D-MB method. Among these five CNN methods, RRBDNet and FFANet result in the highest PSNR and SSIM than the other CNN methods, while FDUNet leads to the lowest values. In Table 1, the last column “# of parameters” refers to the number of parameters in millions (M) used in the CNN models. The model with fewer parameters corresponds to a lightweight model. As can be seen in Table 1, a lightweight model (e.g., RRDBNet (1.59 M) and FFANet (1.51 M)) can perform better than the model with more parameters (e.g., FDUNet (17.50 M)) in terms of PSNR and SSIM, which shows that the CNN model with more parameters does not guarantee better performance. Besides, the models with similar parameters (EDSR (1.52 M), RRDBNet (1.59 M), and FFANet (1.51 M)) result in different performance, and thus, it is important to identify the suitable CNN model based on the performance requirements and the number of parameters required. These five CNN models were further compared using experimental data.

**Fig. 4:**
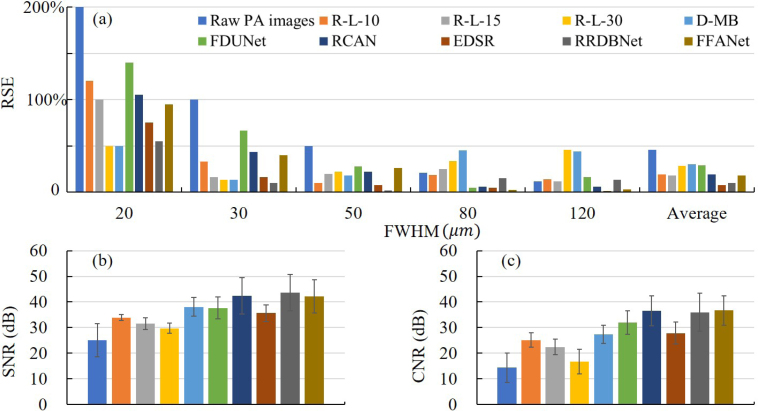
Quantitative analysis for tungsten wire imaging experiment. (a) RSE by different methods. The “Average” bars are the average RSE defined as the average of RSE of cases 2–5 (the original diameters of 30, 50, 80, and 120μm). (b) SNR by different methods. (c) CNR by different methods.

Fig. 3 shows the results using experimentally-acquired AR-PAM images of tungsten wires. Qualitatively, the results by CNN methods (Figs. 3(f)–3(j)) are smoother than the results by traditional methods (Figs. 3(b)–3(e)). Besides, the former shows less noise or artifacts than the latter. Quantitatively, FWHM is extracted from Fig. 3 (FWHM shown in Section 3 of Supplement 1), and RSE is compared in Fig. 4(a).

For the original diameters of 20 μm, 30 μm, 50 μm, 80 μm, and 120 μm, they are denoted as the cases 1–5, respectively. For case 1, all methods have RSE ≥ 50%, which indicates the limitation of resolution enhancement by these deconvolution methods. Therefore, we compare cases 2–5 among these methods in the following. First, for R-L-10, the most accurate deconvolution is realized in case 3 (the original diameter of 50 μm vs. recovered FWHM of 45 μm; RSE of 10%). However, for R-L-15 and R-L-30, the most accurate deconvolution is achieved in case 5 (the original diameter of 120 μm vs. recovered FWHM of 106 μm; RSE of 12%) and case 2 (the original diameter of 30 μm vs. recovered FWHM of 34 μm; RSE of 13%), respectively. This suggests that it is challenging to use R–L deconvolution to recover multiscale object size simultaneously within a certain iteration time. Secondly, for D-MB deconvolution, the most accurate deconvolution is case 2 (the original diameter of 30 μm vs. recovered FWHM of 34 μm; RSE of 13%), while RSE for cases 3–5 is larger (notably cases 4 and 5). Therefore, D-MB deconvolution also fails to simultaneously recover multiscale object size. Thirdly, among the five CNN methods, EDSR and RRDBNet show overall smaller RSE in cases 2–5 (RSE of 2%–17% for EDSR; RSE of 2%–15% for RRDBNet) compared with R–L and D-MB methods, suggesting that the two CNN methods perform well in simultaneously recovering multiscale object size. To quantify the capability of recovering multiscale object size, the average RSE is defined as the average of RSE of cases 2–5, as shown in Fig. 4(a). It can be seen that EDSR and RRDBNet have distinctly smaller average RSE (<10%), while the other methods suffer large average RSE (>18%). In Figs. 4(b) and 4(c), the three CNN methods, RCAN, RRDBNet, and FFANet, achieve better SNR and CNR than the other methods. D-MB, FDUNet, and EDSR have moderate performance in SNR and CNR.

**Fig. 5:**
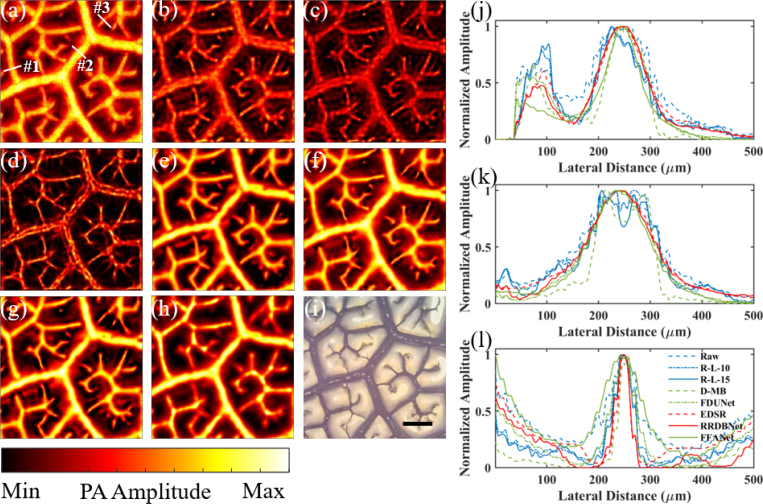
Resolution enhancement enabled by R–L deconvolution, D-MB deconvolution, and the CNN methods for phantom imaging of leaf veins. AR-PAM images: (a) raw PA image, (b) by R-L-10, (c) by R-L-15, (d) by D-MB, (e) by FDUNet, (f) by EDSR, (g) by RRDBNet, and (h) by FFANet. (i) optical microscopy image. (j–l) 1D profiles along the lines #1-#3, respectively, in (a)–(h). Scale bar: 500 μm.

Fig. 5 shows the resolution enhancement for phantom imaging of leaf veins by the deconvolution methods. Fig. 5(a) shows the raw PA image, which presents multiscale line branches. Then, R-L-10 deconvolution, R-L-15 deconvolution, D-MB deconvolution, FDUNet, EDSR, RRDBNet, and FFANet were applied to the raw PA image, and the results are shown in Figs. 5(b)–5(h), respectively. In part due to the relatively poor performance of R-L-30 and RCAN in Fig. 3, Fig. 4, they are excluded in the comparison in Fig. 5. An image of the leaf phantom observed by an optical microscope is shown in Fig. 5(i), which can be regarded as the ground truth. For better comparison, three representative regions are chosen, as indicated by the three lines #1-#3 in Fig. 5(a), to compare leaf vein branch FWHM in Figs. 5(a)–5(h), and the 1D profiles are shown in Figs. 5(j)–5(l), respectively. The branch size in order is: #2 (large) >#1 (middle) >#3 (small). By comparing Figs. 5(a) and 5(i), large branches (e.g., #2) have similar size, while small branches (e.g., #3) are blurred, which confirms the nonlinear relation between $d_{o}$ (Fig. 5(i)) and $d_{c}$ (Fig. 5(a)) in Eq. (3). At first glance, R-L-15 (Fig. 5(c)) leads to smaller feature size than R-L-10 (Fig. 5(b)), which is not surprising. D-MB (Fig. 5(d)) also produces reduced feature size comparable to Fig. 5(c) but suffers severe discontinuities and separation for the large branches (e.g., #2; the branch is divided into two.). By contrast, the results by CNN methods (Figs. 5(e)–5(h)) show better image quality in terms of pattern continuity and smoothness.

Further, the 1D profiles along the three lines #1-#3 are compared. In Fig. 5(j) for the line #1, the FWHM is 117μm, 107 μm, 107 μm, 87 μm, 107 μm, 105 μm, 105 μm, and 78 μm for Figs. 5(a)–5(h), respectively, and the corresponding ground truth is 95 μm (from Fig. 5(i)). The results show that for the representative middle branch size, the CNN (except FFANet), R–L, and D-MB methods can enhance resolution to some degree (RSE < 13%), although D-MB over-processed the raw PA image (i.e., the deconvolved size < the ground truth). In Fig. 5(k) for the line #2, the FWHM is 157 μm, 154 μm, 138 μm, 117 μm, 144 μm, 149 μm, 146 μm, and 127 μm for Figs. 5(a)–5(h), respectively, and the corresponding ground truth is 148 μm (from Fig. 5(i)). The results indicate that for the representative large branch size, the CNN (except FFANet) and R–L methods (RSE < 7%) perform better than D-MB for accurate deconvolution. Finally, in Fig. 5(l) for the line #3, the FWHM is 64 μm, 47 μm, 45 μm, 33 μm, 52 μm, 31 μm, 33 μm, and 88 μm for Figs. 5(a)–5(h), respectively, and the corresponding ground truth is 33 μm (from Fig. 5(i)). The results suggest that for the representative small branch size, the CNN (except FDUNet and FFANet) and D-MB methods (RSE <6%) achieve better results than R–L for deconvolution. Therefore, considering the high-fidelity deconvolution of multiscale line branches, the best performance is achieved by the CNN methods of EDSR and RRDBNet.

**Fig. 6:**
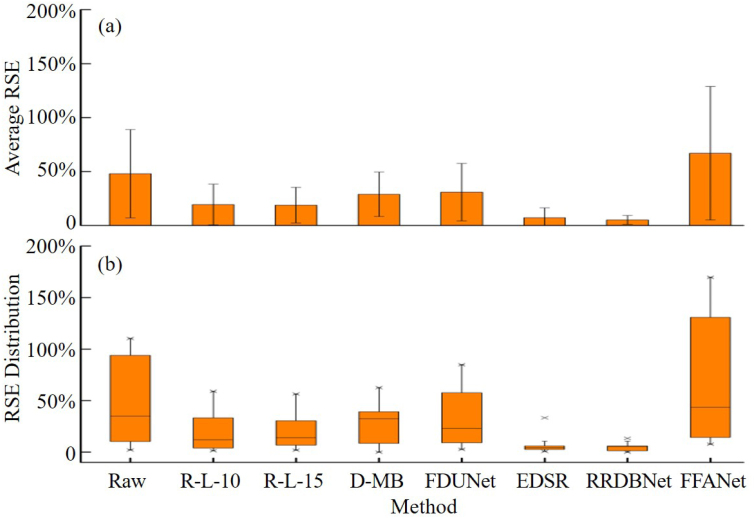
RSE results of the ten regions by different methods. (a) average RSE with the error bars as standard deviations. (b) RSE distribution in boxplots.

To consider more regions besides the lines #1-#3, seven other regions are further selected to compare their 1D profiles (results not shown). The original diameters (from Fig. 5(i)) of the chosen ten regions are, in order, 33 μm (the line #3), 33 μm, 39 μm, 45 μm, 51 μm, 95 μm (the line #1), 105 μm, 143 μm, 148 μm (the line #2), and 155 μm. The selected original feature size varies to some extent (including the size larger and smaller than the PSF), so it can be used to validate accurate deconvolution of multiscale object size. Similarly, the average RSE is defined as the average of RSE of the ten regions. The average RSE by different methods is shown in Fig. 6(a). RSE distribution is also shown in Fig. 6(b). As can be seen, EDSR and RRDBNet perform distinctly better than the other methods, which is consistent with the results in Fig. 5. Interestingly, FFANet performs even worse than raw PA images in terms of average RSE, which is attributed that FFANet fails to recover either the large or small object size.

**Fig. 7:**
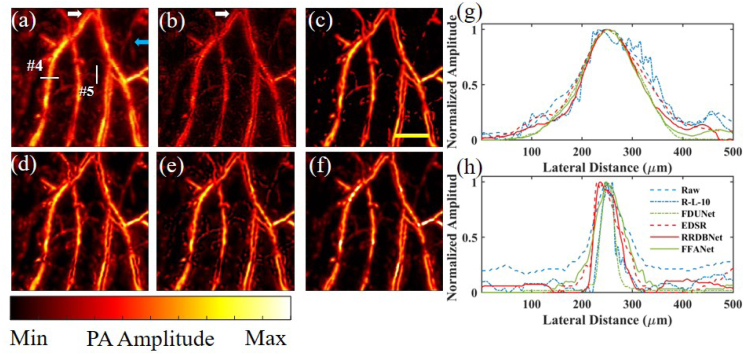
Resolution enhancement results for *in vivo* imaging of mouse ear blood vessels. AR-PAM images: (a) raw PA image, (b) by R-L-10, (c) by FDUNet, (d) by EDSR, (e) by RRDBNet, and (f) by FFANet. (g,h) 1D profiles along the lines #4 (g) and #5 (h), respectively, in (a)–(f). The white arrow indicates the representative area with closely-located arteries and veins. The blue arrow indicates the representative area with small vessels. Scale bar: 1 mm.

Fig. 7 shows the resolution enhancement enabled by different deconvolution methods for the *in vivo* image of mouse ear blood vessels. Fig. 7(a) shows the raw PA image. As can be seen, arteries and veins are closely located (e.g., indicated by the white arrow in Fig. 7(a)) and cannot be well distinguished. Then, R-L-10 deconvolution and the CNN methods were applied to the raw PA image, and the results are shown in Figs. 7(b)–7(f). Similarly, in part due to the poor performance of D-MB and similar performance of R-L-15 (to R-L-10) in Fig. 6, D-MB and R-L-15 are excluded in the comparison in Fig. 7. As shown in Fig. 7(b), although arteries and veins can be more easily distinguished (e.g., indicated by the white arrow in Fig. 7(b)), the discontinuity cannot be completely avoided. By contrast, in Figs. 7(c)–7(f), arteries and veins can not only be easily identified compared with Fig. 7(a), but the pattern continuity is also preserved.

It is also essential to evaluate the high-fidelity recovery of multiscale object size by these methods. Unlike the previous phantom experiments, the actual vessel size (ground truth) cannot be easily obtained by optical microscopy. Alternatively, Fig. 7(a) and Eq. (3) are used to compute the possible original vessel size (i.e., an estimated value). Two representative regions are chosen, as indicated by the two lines #4 and #5 in Fig. 7(a), to compare vessel FWHM in Figs. 7(a)–7(f), and the 1D profiles are shown in Figs. 7(g) and 7(h), respectively. The estimated vessel diameters of the lines #4 and #5 are 145 μm and 51 μm respectively. In Fig. 7(g) for the line #4, the FWHM is 159 μm, 156 μm, 136 μm, 153 μm, 124 μm, and 129 μm for Figs. 7(a)–7(f), respectively. The results show that for relatively large vessels, R-L-10 deconvolution and the CNN method by EDSR achieve similar fidelity in recovering the original vessel size (RSE < 8%). Besides, the RSE by RRDBNet is ∼14%, indicating that RRDBNet still performs well. In Fig. 7(h) for the line #5, the FWHM is 83 μm, 31 μm, 31 μm, 72 μm, 55 μm, and 65 μm for Figs. 7(a)–7(f), respectively. The results suggest that for small vessels that have similar FWHM to the PSF, the CNN method by RRDBNet can accurately recover their original vessel size (RSE < 8%), but R-L-10 and FDUNet over-processed the raw PA image. Note that the RSE by EDSR is ∼41%, indicating EDSR does not perform that well. As for noise level comparison among Figs. 7(b)–7(f), Fig. 7(c) (FDUNet) shows less noise, yet some small vessels are also suppressed. Except FDUNet, when comparing the image contrast of small vessels (e.g., the same vessels in Figs. 7(a)–7(f) indicated by the blue arrow in Fig. 7(a)), Figs. 7(d) (EDSR) and 7(e) (RRDBNet) enable better contrast, while Fig. 7(f) (FFANet) shows relatively low contrast. For quantitative comparison, the SNR and CNR of the same small vessels indicated by the blue arrow in Fig. 7(a) are calculated. The SNR of the vessels is 18.79, 18.56, 37.41, 18.95, 21.56, and 19.32 in Figs. 7(a)–7(f), respectively. For the same vessels, the CNR is 3.52, 2.93, 12.83, 6.45, 6.49, and 3.66 in Figs. 7(a)–7(f), respectively. All CNN methods show better SNR and CNR compared to R-L. Among CNN models, FDUNet obtains the highest SNR and CNR but suffers severe discontinuities (the corresponding area in Fig. 7(c) indicated by the blue arrow in Fig. 7(a)). RRDBNet obtains the second highest SNR and CNR, showing satisfactory recovery of RRDBNet in terms of quantitative metrics. Therefore, based on the above analysis, RRDBNet in Fig. 7(e) realizes the best results in terms of high-fidelity recovery of multiscale vessel size, good continuity, and high contrast for small vessels.

**Fig. 8:**
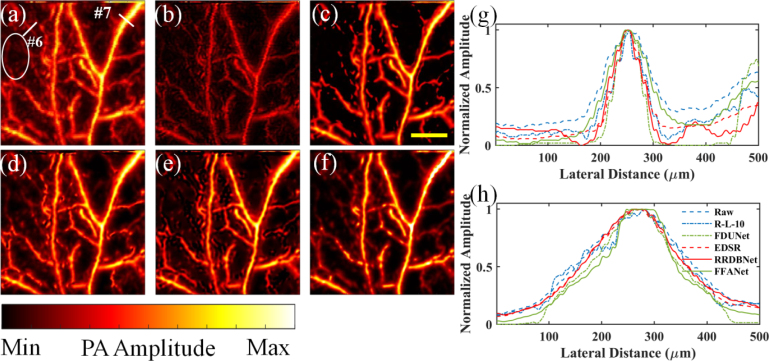
Resolution enhancement results for *in vivo* imaging of rat ear blood vessels. AR-PAM images: (a) raw PA image, (b) by R-L-10, (c) by FDUNet, (d) by EDSR, (e) by RRDBNet, and (f) by FFANet. (g,h) 1D profiles along the lines #6 (g) and #7 (h), respectively, in (a)–(f). The blue arrow indicates the representative area with small vessels. Scale bar: 1 mm.

For further demonstration, resolution enhancement by different deconvolution methods for the *in vivo* image of rat ear blood vessels was investigated in Fig. 8. Compared with Fig. 7(a), Fig. 8(a) has a higher vessel density. Similarly, among Figs. 8(b)–8(f), Fig. 8(c) (FDUNet) has the darkest background, yet some small vessels disappear. For small vessels (e.g., indicated by the blue arrow in Fig. 8(a)), Figs. 8(d) (EDSR) and 8(e) (RRDBNet) preserve more small features.

Quantitatively, two representative regions are chosen, as indicated by the two lines #6 and #7 in Fig. 8(a), to compare vessel FWHM in Figs. 8(a)–8(d), and the 1D profiles are shown in Figs. 8(g) and 8(h), respectively. Similar to Fig. 7, by Fig. 8(a) and Eq. (3), the estimated vessel diameters of the lines #6 and #7 is 85 μm and 214 μm, respectively. In Fig. 8(g) for the line #6, the FWHM is 107 μm, 68 μm, 64 μm, 65 μm, 75 μm, and 90 μm in Figs. 8(a)–8(f), respectively. The results show that for relatively small vessels, RRDBNet and FFANet perform better in high-fidelity size recovery (RSE < 12%). In Fig. 8(h) for the line #7, the FWHM is 224 μm, 206 μm, 177 μm, 204 μm, 207 μm, and 164 μm in Figs. 8(a)–8(d), respectively. The results show that for relatively large vessels, R-L-10, EDSR, and RRDBNet realize better size recovery (RSE < 5%). Therefore, similar to Figs. 7, RRDBNet achieves the most accurate deconvolution of multiscale vessel size. Similarly, the SNR and CNR of the same small vessels indicated by the blue arrow in Fig. 8(a) are calculated. The SNR for the vessels is 21.84, 21.24, 27.97, 38.83, 35.08, and 50.85 in Figs. 8(a)–8(f), respectively. For the same vessels, the CNR is 12.24, 7.77, 17.03, 30.47, 23.44, and 39.22 in Figs. 8(a)–8(f), respectively. Similar to Fig. 7, all CNN methods show better results compared to R-L. Both the SNR and CNR in order are: FFANet > EDSR > RRDBNet > FDUNet > R-L-10. Although not the highest, RRDBNet shows decent results in terms of SNR and CNR. Therefore, considering the overall performance including multiscale recovery, pattern continuity, and SNR and CNR of small vessels, RRDBNet would still be a better choice.

## 4 Discussion

In this work, the CNN-based deconvolution technique was investigated for resolution enhancement in AR-PAM images. The novelty of this work lies in three aspects. First, the CNN method was applied to improve the AR-PAM image resolution in the focal region for the first time. Secondly, a strategy for generating training data using simulated data is proposed, which circumvents the difficulty of obtaining real high-resolution AR-PAM images considering that it would be challenging to build an AR-PAM system with lateral resolution less than half of 65 μm. Thirdly, five different CNN models were evaluated, and two CNN models demonstrated better recovery of multiscale feature size compared with the traditional methods and the other three CNN methods.

The accurate deconvolution of multiscale object size enabled by the CNN method can be explained as follows. First, each single convolution layer deals with local features in fixed size of a region of interest (ROI), and different convolution layers can handle the features in different size of ROIs. The stacked convolution layers with nonlinear activation ensure the CNN can distinguish the characteristics among different feature size in AR-PAM images and learn the nonlinearity of Eq. (3). Secondly, training with a large amount of data ensures that the parameters of CNN can be learned successfully without overfitting. Besides, the great performance by the CNN method may be partly attributed to the fact that the CNN method can learn to be robust to noise, which is inevitable in *in vivo* AR-PAM images. By contrast, the R-L method is less robust to the noise, which simply degrades the performance. As can be seen in Fig. 3, the CNN results (Figs. 3(f)–3(j)) present less noise compared with R-L results (Figs. 3(b)–3(d)).

Further, the deconvolution performance was compared among the five CNN methods. Overall, EDSR and RRDBNet outperformed the other CNN models in terms of high-fidelity recovery of multiscale feature size. Although EDSR, RRDBNet, and FFANet are lightweight models compared with FDUNet and RCAN, the former (EDSR, RRDBNet, and FFANet) produced higher PSNR and SSIM (Table 1) in the testing set of simulated data. As for FDUNet, it features an encoder–decoder model structure, which is different from the other CNN models. As can be seen in Figs. 7(c) and 8(c), FDUNet suffers discontinuity for small features (e.g., the blue arrows in Figs. 7(c) and 8(c)), and the results seem to be less reliable due to distinct PA amplitude between the signal and background regions. This may be explained by the use of downsampling layers, leading to the information loss of small features with limited pixels. For the remaining four models, they were built using the global residual learning approach without downsampling layers. They obtained better performance than FDUNet (e.g., Fig. 4(a)), which suggests the effectiveness of the approach. When further comparing the four models (i.e., excluding FDUNet), EDSR and RRDBNet only used residual connections or residual dense connections, which proved to be more effective (e.g., Figs. 4(a) and 6). By contrast, RCAN used the design of channel attention, and FFANet used the design of both channel attention and pixel attention. The poor performance on the experimental data by RCAN and FFANet suggests that the design of channel attention can cause the overfitting to the training set of the simulated data and therefore exhibits poor generalization for the experimental data (e.g., poor performance of FFANet in the average RSE in Figs. 4(a) and 6 compared with EDSR and RRDBNet). Finally, for the comparison between EDSR and RRDBNet, EDSR performed slightly better than RRDBNet in phantom images of tungsten wires (e.g., Fig. 4(a)), while RRDBNet performed better than EDSR in *in vivo* images in terms of high-fidelity recovery of multiscale vessel size (Fig. 7, Fig. 8), as detailed previously. This may be because RRDBNet is more robust to noise than EDSR. Therefore, RRDBNet would be a better choice for *in vivo* images that typically have limited SNR.

In this study, speckle noise was added to the low-resolution image for the simulated data. Although it was reported that PA imaging has the speckle-free nature [29], another study mentioned that speckle noise exists and comes from acoustically inhomogeneous tissue in PA imaging [30]. In our demonstrations, phantom images may have little speckle noise, but in vivo images are expected to have speckle noise to some degree due to acoustically inhomogeneous tissue. Besides, adding speckle noise for the simulated data would improve the generalization ability of the CNN models, which can be used for the cases of little and high speckle noise.

In our demonstration of phantom imaging, the original diameter of cylindrical objects was used to approximate $d_{o}$, which is defined as the FWHM of the ground truth PA image in Eq. (3). That is, the original diameter of cylindrical objects and the FWHM of the ground truth PA image are not exactly the same. The approximation is reasonable, as explained as follows. First, because both tungsten wires and leaf veins are strong light absorbers, the light absorption mainly occurs at the top surface of these cylindrical objects, and thus, the absorption profile can be modeled as rectangular profiles. Secondly, as shown in Section 4 of Supplement 1, the difference between the convolved FWHM ($d_{c}$) from a Gaussian PSF (with FWHM of $d_{p}$ and a *Gaussian profile (with FWHM of* $d_{o}$) and that from the same Gaussian PSF and a *rectangular profile (with a diameter of* $d_{o}$) is small compared with most RSE values in this study (see Figs. 4(a) and 6).

Currently, the simulated data are used as the training data, which were generated using the PSF with FWHM of 65μm. The current training data cannot be directly used as the training data for other AR-PAM systems with different size of PSF. Fortunately, new training data can be easily generated simply by changing the corresponding PSF of the AR-PAM system, which demonstrates the advantage of our methodology in easy adaptation to different AR-PAM systems. Besides, acquiring experimental data for training is also meaningful to explore. However, there are a few challenges. First, as mentioned previously, building an AR-PAM system with higher lateral resolution is not easy, and the performance can be sacrificed (e.g., using a high-frequency acoustic transducer at the expense of the penetration depth). Secondly, it is time-consuming to collect enough image pairs (paired low-resolution and high-resolution (i.e., ground truth) AR-PAM images) for training. In the future, it is worth trying to use experimentally-acquired data for training though. Alternatively, a cycle generative adversarial network approach may be used [31], which circumvents the experimental acquisition of high-resolution AR-PAM images. Briefly, simulated ground truth and simulated low-resolution image pairs (paired data) in conjunction with experimental low-resolution images (i.e., unpaired data) can be generated and experimentally acquired, and finally, experimental high-resolution images (corresponding to the experimental low-resolution images) could be generated to obtain paired data for training.

In our demonstrations, the CNN method was applied to AR-PAM images acquired around the focal plane. For AR-PAM images acquired in the out-of-focus region, a synthetic aperture focusing technique (SAFT) to restore the lateral resolution can be applied followed by the CNN method to further improve the resolution, which is a two-step processing approach for resolution enhancement [5], [15]. Besides, it would be possible that the CNN method could learn to process focal and out-of-focus data as well, reducing the number of processing steps. In this regard, for out-of-focus data, the CNN model may be developed to incorporate the processing similar to combined SAFT (e.g., [24]) and deconvolution. Currently, the proposed method cannot improve axial resolution as only two-dimensional (2D) lateral images are processed. It would be possible that the CNN method can be used to improve axial resolution. One approach is to process three-dimensional (3D) images with a 3D CNN model. In this case, a 3D PSF incorporating axial resolution should also be adopted. Another approach is to conduct a two-step processing based on the independence between lateral resolution and axial resolution [5], [15]), one CNN model for 2D lateral deconvolution and the other CNN model for 1D axial deconvolution. That is, the two CNN models are used sequentially, and they are trained separately.

The performance of the CNN method may be further improved by optimizing the model structure and the training data. As shown in this study, different CNN methods present different recovery performance, so the model structure can be tailored according to specific features.

## 5 Conclusions

In this work, we investigated multiscale deconvolution in AR-PAM. A data-driven CNN method to learn the prior knowledge of the AR-PAM system was developed and tested. Five different CNN models were implemented, and conventional deconvolution (R-L and D-MB) and CNN deconvolution methods were compared through both phantom and *in vivo* experiments. Among all deconvolution methods, two CNN models (EDSR and RRDBNet) achieved excellent performance. Notably, when using RRDBNet for *in vivo* images of blood vessels, high-fidelity recovery of multiscale vessel size, good continuity, and high contrast for small vessels were realized. Our work is promising to enhance resolution for multiscale microvascular AR-PAM images. The methodology may be extended and applied to other imaging modalities with resolution limited by the PSF, such as OR-PAM and fluorescence imaging, for high-fidelity deconvolution.

## Declaration of Competing Interest

The authors declare that they have no known competing financial interests or personal relationships that could have appeared to influence the work reported in this paper.

## Appendix A Supplementary data

MMC S1 .
