# Supplementary material for: High-fidelity deconvolution for acoustic-resolution photoacoustic microscopy enabled by convolutional neural networks
Source: Photoacoustics. 2022 Apr 26;26:100360. doi: 10.1016/j.pacs.2022.100360 (PMC9095893; doi:10.1016/j.pacs.2022.100360)
Supplement: MMC S1 — . [file mmc1.pdf]

supplement1 Click here to view linked References

# High-fidelity deconvolution for acoustic-resolution photoacoustic microscopy enabled by convolutional neural networks: Supplemental document

This document provides supplementary information to "High-fidelity deconvolution for acoustic-resolution photoacoustic microscopy enabled by convolutional neural networks" regarding evaluation metrics, CNN architectures and training details, FWHM extracted from Fig. 3, and comparison of the convolved FWHM using different $d_o$.

## 1. Evaluation metrics

Two metrics of PSNR and SSIM are used to evaluate image recovery performance. PSNR is expressed as:

$$\mathrm{PSNR} = 20\log(\frac{255}{\sqrt{MSE}})$$

$$MSE = \frac{1}{MN}\sum_{m,n}^{M,N}(o_{mn} - o'_{mn})^2,$$

where $o_{mn}$ and $o'_{mn}$ are the pixel values of the ground truth PA image and predicted PA image on the m<sup>th</sup> row and n<sup>th</sup> column (with size $M\times N$), respectively. SSIM is defined as:

$$SSIM = \frac{(2\mu_o\mu_{o'}+2.55^2)(2\sigma_{o,o'}+7.65^2)}{(\mu_o{}^2+\mu_{o'}{}^2+2.55^2)(\sigma_o{}^2+\sigma_{o'}{}^2+7.65^2)},$$

where $\mu_o$ and $\mu_{o'}$ are the means of the ground truth and predicted PA images, respectively; $\sigma_o$ and $\sigma_{o'}$ are the standard deviations of the ground truth and predicted PA images, respectively, and $\sigma_{o,o'}$ is the covariance between the ground truth and predicted PA images.

Quantitative metrics of SNR and CNR, which are calculated using the following equations, are also used to evaluate noise level and image contrast, respectively.

$$SNR = 20\log(\frac{\max(I)}{\sigma})$$

$$CNR = \frac{1}{n}\sum_{i=1}^{n}\left[20\log\left(\frac{\mu_i - \mu_b}{\sigma}\right)\right],$$

where $I$ is the selected region of interest (signal) and $\sigma$ is the standard deviation of the background (noise); $\mu_i$ and $\mu_b$ are the mean values of the selected region of interest (signal) and the background (noise), respectively.

## 2. CNN architectures and training details

Five CNNs are used for the model training, which are FDUNet, RCAN, EDSR, RRDBNet, and FFANet. The FDUNet architecture refers to the previous work [S1], and no modification is made. The RCAN architecture refers to the previous work [S2], and two residual groups are used. In each residual group, there are 20 residual channel attention blocks. The EDSR refers to the previous work [S3], and 16 residual connection modules are used. The RRDBNet refers to the previous work [S4], and two residual in residual dense blocks are used. The FFANet refers to the previous work [S5], and three groups of feature attention

modules are used. For each group, there are six basic blocks composed of skip connections and feature attention modules. More details on the design of the basic block can be found in previous work [S5].

For model training, the model was trained with a batch size of 2, and a total of 200000 batches were trained. The initial learning rate is 0.0001. Each of the five CNN models was trained for 800 epochs, and no early stopping was applied. For each CNN model, no hyperparameters were tuned, and no extra model selection was performed. The training data and testing data were generated using the same procedure (Section "Methods" in main text about simulated data). Rotation and noise level addition were randomly applied to each simulated image for data augmentation. The augmented images from the same simulated image (before data augmentation) were all used either in the training dataset or in the testing dataset. That is, the training dataset and testing dataset are independent to ensure that the performance evaluation is valid.

## 3. FWHM extracted from Fig. 3

Table S1. FWHM extracted from Fig. 3 (Units: μm).

| (a) Raw images | (b) R-L-10 | (c) R-L-15 | (d) R-L-30 | (e) D-MB | (f) FDUNet | (g) RCAN | (h) EDSR | (i) RRDBNet | (j) FFANet |
|---|---|---|---|---|---|---|---|---|---|
| 60 | 44 | 40 | 30 | 30 | 48 | 41 | 35 | 31 | 39 |
| 60 | 40 | 35 | 34 | 34 | 50 | 43 | 35 | 33 | 42 |
| 75 | 45 | 40 | 39 | 41 | 64 | 61 | 54 | 51 | 63 |
| 97 | 65 | 60 | 53 | 44 | 76 | 85 | 76 | 68 | 78 |
| 134 | 106 | 106 | 65 | 67 | 100 | 113 | 122 | 104 | 116 |

## 4. Comparison of the convolved FWHM using different $d_o$

Fig. S1(a) shows a Gaussian profile with FWHM of $d_o$, while Fig. S1(b) shows a rectangular profile with a diameter (i.e., width of the profile) of $d_o$. A Gaussian PSF with FWHM ($d_p$) of 65 μm is assumed. The difference between the convolved FWHM ($d_c$) from the PSF and Fig. S1(a) and the convolved FWHM from the same PSF and Fig. S1(b) is compared. The results are shown in Table S2. As can be seen, the difference is relatively small (from −2.35% to −12.5%).

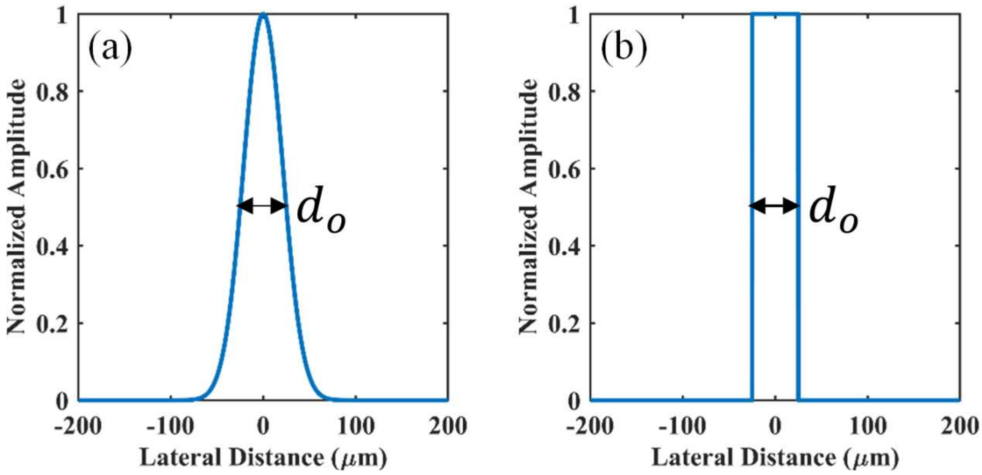


Fig. S1. Illustration of (a) a Gaussian (with FWHM of $d_o$) profile and (b) a rectangular (with a diameter of $d_o$) profile.

Table S2. The convolved FWHM (Units: μm).

| Gaussian PSF ($d_p$) | FWHM or diameter ($d_o$)[a] | Convolved FWHM ($d_c$) of $d_p \otimes d_o$ | | Difference between the two convolved FWHM[a] |
|---|---|---|---|---|
| | | When $d_o$ is a Gaussian profile (Fig. S1(a)) | When $d_o$ is a rectangular profile (Fig. S1(b)) | |
| 65 | 20 | 68.0 | 66.4 | –2.35% |
| 65 | 30 | 71.6 | 68.2 | –4.75% |
| 65 | 50 | 82.0 | 74.4 | –9.27% |
| 65 | 80 | 103.1 | 90.2 | –12.5% |
| 65 | 120 | 136.5 | 122.2 | –10.5% |
| 65 | 200 | 210.3 | 200.2 | –4.80% |

[a]Calculated by [(the value in column 4)–(the value in column 3)]/(the value in column 3)*100%

## References


[S1] A. Sharma and M. Pramanik, "Convolutional neural network for resolution enhancement and noise reduction in acoustic resolution photoacoustic microscopy," *Biomed. Opt. Express,* vol. 11, no. 12, pp. 6826-6839, 2020.

[S2] Y. Zhang, K. Li, K. Li, L. Wang, B. Zhong, and Y. Fu, "Image Super-Resolution Using Very Deep Residual Channel Attention Networks," in *ECCV 2018*, pp. 294-310.

[S3] B. Lim, S. Son, H. Kim, S. Nah, and K. M. Lee, "Enhanced Deep Residual Networks for Single Image Super-Resolution," in *2017 IEEE Conference on Computer Vision and Pattern Recognition Workshops (CVPRW)*, 21-26 July 2017.

[S4] X. Wang, K. Yu, S. Wu, J. Gu, Y. Liu, C. Dong, Y. Qiao, and C. C. Loy, "ESRGAN: Enhanced Super-Resolution Generative Adversarial Networks," in *ECCV 2018 Workshops*, 63-79, 2019.

[S5] X. Qin, Z. L. Wang, Y. C. Bai, X. D. Xie, H. Z. Jia, and I. Assoc Advancement Artificial, "FFA-Net: Feature Fusion Attention Network for Single Image Dehazing," in AAAI Conference on Artificial Intelligence, vol. 34, pp. 11908-11915, 2020.
